# A monoclonal antibody activating AdipoR for type 2 diabetes and nonalcoholic steatohepatitis

**DOI:** 10.1126/sciadv.adg4216

**Published:** 2023-11-10

**Authors:** Naomi Asahara, Miki Okada-Iwabu, Masato Iwabu, Kouichi Wada, Kozo Oka, Toshimasa Yamauchi, Takashi Kadowaki

**Affiliations:** ^1^ Sohyaku. Innovative Research Division, Mitsubishi Tanabe Pharma Corporation, 1000, Kamoshida-cho, Aoba-ku, Yokohama 227-0033, Japan.; ^2^ Department of Diabetes and Metabolic Diseases, Graduate School of Medicine, The University of Tokyo, Tokyo 113-0033, Japan.; ^3^ Laboratory for Advanced Research on Pathophysiology of Metabolic Diseases, The University of Tokyo, 7-3-1 Hongo, Bunkyo-ku, Tokyo 113-0033, Japan.; ^4^ Department of Endocrinology, Metabolism and Nephrology, Graduate School of Medicine, Nippon Medical School, Tokyo 113-8603, Japan.; ^5^ Sohyaku. Innovative Research Division, Mitsubishi Tanabe Pharma Corporation, Shonan Health Innovation Park, 2-26-1, Muraoka-Higashi, Fujisawa, Kanagawa 251-8555, Japan.; ^6^ Toranomon Hospital, 2-2-2 Toranomon, Minato-ku, Tokyo 105-8470, Japan.

## Abstract

Adiponectin receptors, AdipoR1 and AdipoR2 are promising targets for the prevention and treatment of metabolic diseases. In this study, we aimed to establish agonistic antibodies against AdipoR1 and AdipoR2 with a long enough half-life to provide a means of improving poor medication adherence associated with preclinical small-molecule AdipoR agonists or existing antidiabetic drugs. Monoclonal antibodies were obtained by immunizing AdipoR knockout mice with human AdipoR-expressing cells. Of the antibodies shown to bind to both, an agonist antibody was obtained, which exhibited adenosine 5′-monophosphate–activated protein kinase–activating properties such as adiponectin and was named AdipoR-activating monoclonal antibody (AdipoRaMab). AdipoRaMab ameliorated glucose intolerance in high-fat diet–fed mice, which was not observed in AdipoR1·AdipoR2 double knockout mice. AdipoRaMab exhibited anti-inflammatory and antifibrotic effects in the nonalcoholic steatohepatitis (NASH) model, indicating its therapeutic potential in diabetes and in NASH. In addition, the results of this study indicated that AdipoRaMab may exert therapeutic effects even in a once-monthly dosing regimen through its humanization.

## Teaser

The AdipoR-activating antibody has the potential to exert therapeutic effects on diabetes and NASH even in a once-monthly dosing.

## INTRODUCTION

While adiponectin (*1*–*4*) is produced mainly by adipocytes and is involved in glucose metabolism, lipid metabolism, and vascular endothelial function, its plasma levels are reduced in obesity and type 2 diabetes (*5*). In a mouse model of obesity/type 2 diabetes, a decreased plasma adiponectin concentration causes insulin resistance and dyslipidemia, while adiponectin administration improves these conditions at appropriate doses (*6*). The mechanism of action of adiponectin has been shown to involve adenosine 5′-monophosphate (AMP)–activated protein kinase (AMPK) activation (*7*–*9*) and peroxisome proliferator–activated receptor-α (PPARα) (*10*, *11*) in the skeletal muscle and liver.

After the adiponectin receptor (AdipoR) was identified in 2003 (*11*), the effects of AdipoR deficiency and knockdown have been examined in mice, and, as a consequence, many of the effects of adiponectin were shown to be mediated by AdipoR (*12*). The crystal structures of human AdipoR1 and AdipoR2 have also been reported (*13*), and the relationship between the crystal structure of AdipoR1 and its downstream signaling has been investigated in recent years (*14*). Further, this has led to a therapeutic approach mimicking the effects of adiponectin. A small-molecule agonist of AdipoR, i.e., AdipoRon, was developed and shown to exhibit the previously reported effects of adiponectin in vitro and in vivo (*15*, *16*). On the other hand, in addition to the high efficacy and safety of antibody formulations, commercial mass production technology has expanded the horizons of their application to diseases that were mainly treated by oral drugs. Several oral agents have been approved for the treatment of type 2 diabetes and provide a wide range of treatment options but are often found insufficient for glycemic control, event control, and safety, and low medication adherence is cited among the reasons for their insufficiency (*17*, *18*). Thus, issues remain. We propose that antibody drugs with long half-lives may address some of these issues, given their enhanced therapeutic effects. AdipoR is a seven-transmembrane receptor with a different orientation from that of the G protein–coupled receptor (GPCR) (*11*). Despite advances in technology for obtaining antibodies that bind to GPCRs, however, to date, only two have been approved as antibody drugs, and no functional monoclonal antibodies have been approved that directly control signaling.

Nonalcoholic steatohepatitis (NASH) is a chronic liver disease associated with inflamed fatty liver, cellular ballooning, and fibrosis and is considered a hepatic manifestation of the metabolic syndrome because it is shown to be strongly associated with dyslipidemia, obesity, hypertension, and insulin resistance (*19*). Moreover, progression of NASH leads to cirrhosis and liver cancer (*20*–*22*). Thus, therapeutic intervention in NASH is called for to prevent the development of liver cancer and cirrhosis. However, no effective therapy is currently available for the treatment of NASH. In NASH, a decrease in plasma adiponectin levels has been suggested to be a NASH-associated factor independent of insulin resistance (*23*, *24*). Furthermore, increased oxidative stress, infiltration of inflammatory cells, and fibrosis have also been reported in NASH animal models with reduced adiponectin levels, while increased expression of adiponectin in the methionine-choline deficiency model has been shown to lead to the suppression of these events (*25*), suggesting that decreased levels of adiponectin may be involved in the pathogenesis of NASH.

In this study, we aimed to develop an AdipoR-activating monoclonal antibody (AdipoRaMab) with immunoglobulin G (IgG) that has a long enough half-life for monthly administration. In addition, we examined whether AdipoRaMab could represent a candidate drug for NASH treatment. The preparation of an agonistic IgG antibody against AdipoR has not been reported to date.

## RESULTS

### Preparation of antibodies recognizing AdipoR

First, efforts were focused on obtaining antibodies recognizing AdipoR using the animal immunity method. To obtain a functional agonist antibody, we considered it important to use an antigen with a native structure and to adopt an antibody screening system that recognized three-dimensional structures. Therefore, a cell from the same species (mouse) as an immunized animal, i.e., an NS0 cell, was used as a host cell, and AdipoR1- or AdipoR2-expressing cells were prepared and used as an immunogen. In addition, given that AdipoR1 and AdipoR2 have high interspecies homology of 98%, which makes it difficult to produce antibodies in normal animals, AdipoR1 knockout (R1 KO), AdipoR2 knockout (R2 KO), or AdipoR1 and AdipoR2 double knockout (R1·R2 DKO) mice were also adopted as immunized animals. Plasma antibody titer determination and antibody productional hybridoma screening were performed using cells expressing AdipoR1 or AdipoR2, as indicated by binding in flow cytometry. To exclude antibodies against NS0 cells, AdipoR-expressing cells were generated using Chinese hamster ovary (CHO) cells of another type for use in flow cytometry.

Hybridomas were prepared from all animals with elevated antibody titers. The monoclonal hybridomas were prepared using the limiting dilution method, and their culture supernatants were evaluated by flow cytometry for binding to AdipoR1- and AdipoR2-expressing CHO versus CHO cells. Positive binding was defined as a histogram shown to have been shifted to a higher fluorescence intensity than that for the fluorescently labeled secondary antibody without the addition of the culture supernatant. As a result, 17 antibody-producing clones binding to AdipoR-expressing cells were obtained. Purified antibodies were prepared from culture supernatants of these hybridomas, and the heavy chain isotypes and light chain subclasses were examined using an isotyping kit (table S1). Subsequently, the binding activity of the purified antibody was evaluated by flow cytometry, and if a histogram shift was detected relative to the isotype control antibody, then the antibody was considered bound (table S1 and fig. S1). Thus, antibodies binding to both AdipoR1 and AdipoR2 were obtained. In addition, a further antibody binding to AdipoR1 alone was also obtained. Most of the AdipoR antibodies thus obtained were shown to be IgM antibodies, with only four being IgG antibodies.

### Acquisition of an AdipoR agonist antibody

**Fig. 1.**
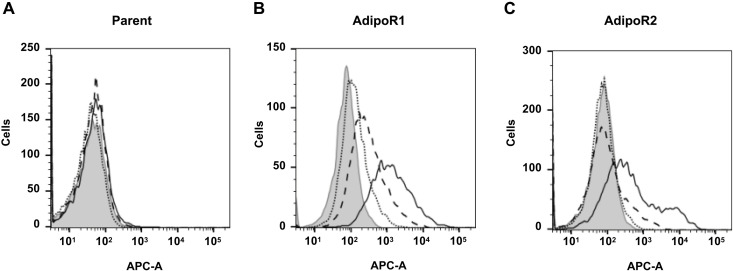
AdipoRaMab bound to AdipoR1 and AdipoR2. AdipoRaMab was evaluated for binding to AdipoR1 or AdipoR2 in AdipoR1- or AdipoR2-expressing cells or their parent strain using flow cytometry, and the results are presented as histograms [Parent strain (**A**), AdipoR1-expressing cells (**B**), AdipoR2-expressing cells (**C**)]. Solid histograms represent results for isotype control monoclonal antibody (Mab) (10 μg/ml). Solid line denotes AdipoRaMab (10 μg/ml), dashed line denotes AdipoRaMab (3 μg/ml), and dotted line denotes AdipoRaMab (1 μg/ml). APC-A, Allophycocyanin area.

**Fig. 2.**
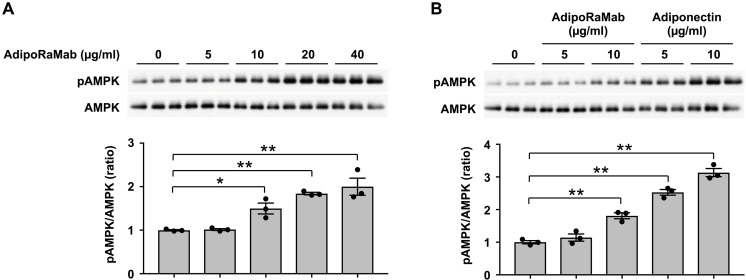
AdipoRaMab increased AMPK phosphorylation at Thr^172^ in C2C12 myotubes. Phosphorylation and amount of AMPK in C2C12 myotubes after myogenic differentiation. C2C12 myotubes were treated (**A**) with or without AdipoRaMab, (**B**) with or without AdipoRaMab, or recombinant human adiponectin at the concentration as indicated for 10 min. AMPK phosphorylation was calculated at Thr^172^ as the ratio of the expression of phosphorylated AMPKα (pAMPK) to that of AMPKα and quantified by Western blotting. Results are expressed as ratios without AdipoRaMab. All values are presented as means ± SEM. *n* = 3. *P < 0.05 and **P < 0.01 compared with no AdipoRaMab [analysis of variance (ANOVA) followed by the Dunnett multiple comparison test].

AMPK activation (Thr^172^ phosphorylation), as mediated by adiponectin signaling (*7*, *8*), was evaluated in C2C12 myotubes after their myogenic differentiation using AdipoR antibodies. AMPK activation was indexed by the ratio of phosphorylated AMPK to AMPK calculated using AMPK and phosphorylated AMPK expression quantified by Western blotting. AdipoR antibodies were considered active if they showed a statistically significantly greater phosphorylated AMPK/AMPK ratio than isotype control antibodies. As a result, AMPK activation was confirmed in five clones (table S1 and fig. S2). Of these, the IgG antibody exhibiting AMPK-activating properties, named AdipoRaMab, was shown to be able to bind to both AdipoR1 and AdipoR2 (Fig. 1). AdipoRaMab increased AMPK phosphorylation at concentrations of 10 to 40 μg/ml in a dose-dependent manner (Fig. 2A and fig. S3), with its AMPK-activating effect similar to that of adiponectin (Fig. 2B and fig. S3). AdipoRaMab did not significantly increase p38MAPK phosphorylation at concentrations that significantly increased AMPK phosphorylation in C2C12 myotubes (fig. S4). These results suggest that AdipoRaMab activates AMPK without involving p38MAPK activation.

### AdipoRaMab ameliorates glucose intolerance via AdipoR

**Fig. 3.**
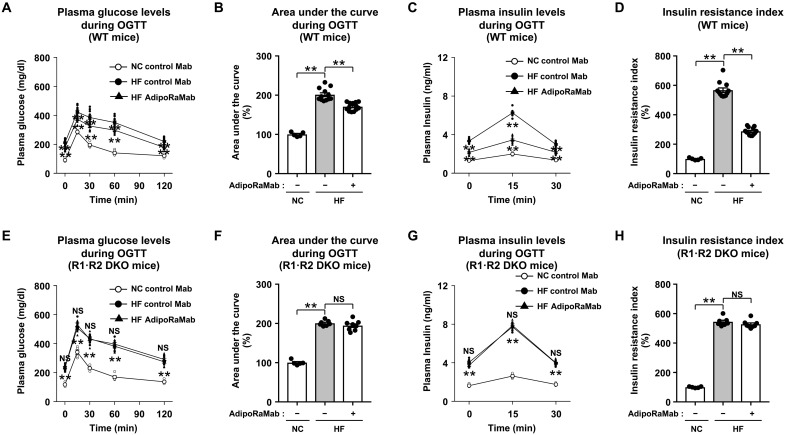
AdipoRaMab improved glucose tolerance and insulin resistance index via AdipoR in high-fat diet–fed mice. Plasma glucose level (**A** and **E**), AUC (**B** and **F**), plasma insulin level (**C** and **G**), and insulin resistance index (**D** and **H**) during the OGTT (1.0 g glucose/kg body weight) in normal chow (NC)– or high-fat (HF) diet–fed, wild-type (WT), and AdipoR1 and AdipoR2 double knockout (R1·R2 DKO) mice, treated four times with AdipoRaMab or isotype control Mab at a once-weekly dose of 10 mg/kg body weight from 4 weeks after initiation of an HF diet onward. AdipoRaMab (−), isotype control Mab treatment. Results in (B), (D), (F), and (H) are expressed as ratios in normal chow–fed mice treated with isotype control Mab. All values are presented as means ± SEM. NC-fed WT or R1·R2 DKO mice treated with isotype control Mab (*n* = 5). HF diet–fed WT mice treated with isotype control Mab or AdipoRaMab (*n* = 12 or 13). HF diet–fed R1·R2 DKO mice treated with isotype control Mab or AdipoRaMab (*n* = 7 or 8). **P < 0.01 compared with isotype control Mab or as indicated (ANOVA followed by the Tukey-Kramer multiple comparison test). NS, not significant.

AdipoRaMab was evaluated for its therapeutic potential against insulin resistance and glucose intolerance using mice with high-fat diet–induced obesity. AdipoRaMab or an isotype control antibody (control antibody; Medical & Biological Laboratories Co. Ltd) was administered intraperitoneally at a once-weekly dose of 10 mg/kg body weight for a total of four doses. An oral glucose tolerance test (OGTT) was performed 1 week after the fourth dosing. Glucose levels were significantly higher in high-fat diet–fed, wild-type mice in the fasting state and at each time point measured during the OGTT, as well as in the area under the curve (AUC) analysis (Fig. 3, A and B). High-fat feeding also resulted in significantly higher insulin levels in the mice in the fasting state and at various time points measured during the OGTT (Fig. 3C), as well as in the calculated insulin resistance index using glucose and insulin during the OGTT (Fig. 3D). In this glucose-intolerant and insulin-resistant mouse model, treatment with AdipoRaMab significantly decreased glucose levels compared to isotype control antibody treatment in the fasting state and at multiple time points measured during the OGTT, as well as in the AUC analysis (Fig. 3, A and B). AdipoRaMab treatment also significantly reduced insulin levels in the fasting state and during the OGTT, as well as in the insulin resistance index (Fig. 3, C and D). The percent reduction in insulin resistance index was approximately 50%. In contrast, these effects were not observed in the R1·R2 DKO mice (Fig. 3, E to H), confirming that these effects were mediated by AdipoR1 and AdipoR2.

**Fig. 4.**
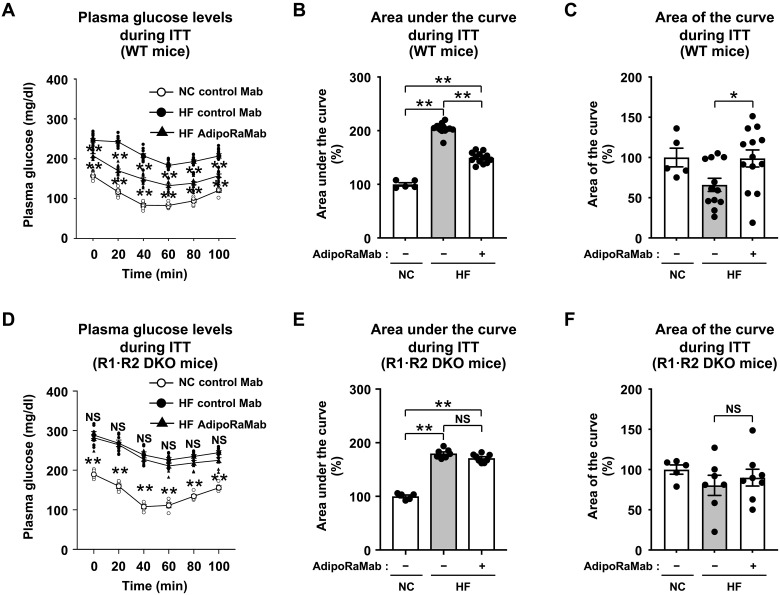
AdipoRaMab improved insulin tolerance via AdipoR in high-fat diet–fed mice. Plasma glucose level (**A** and **D**), AUC (**B** and **E**), and AOC (**C** and **F**) during the ITT (0.5 U insulin/kg body weight) in NC- or HF diet–fed WT and R1·R2 DKO mice, treated four times with AdipoRaMab or isotype control Mab at a once-weekly dose of 10 mg/kg body weight from 4 weeks after initiation of an HF diet onward. AdipoRaMab (−), isotype control Mab treatment. Results in (B), (C), (E), and (F) are expressed as ratios in normal chow–fed mice treated with isotype control Mab. All values are presented as means ± SEM. NC-fed WT or R1·R2 DKO mice treated with isotype control Mab (*n* = 5). HF diet–fed WT mice treated with isotype control Mab or AdipoRaMab (*n* = 12 or 13). HF diet–fed R1·R2 DKO mice treated with isotype control Mab or AdipoRaMab (*n* = 7 or 8). **P < 0.01 compared with isotype control Mab or as indicated (ANOVA followed by the Tukey-Kramer multiple comparison test).

With reference to previous reports (*26*) on adiponectin, we hypothesized that AdipoRaMab counteracts insulin resistance by improving insulin sensitivity and investigated the plasma glucose–lowering effect of AdipoRaMab in an insulin tolerance test (ITT). Glucose levels were significantly higher after insulin administration in high-fat diet–fed, wild-type mice than in normal chow–fed mice at each time point during the ITT and in the AUC analysis (Fig. 4, A and B), suggesting that decreased insulin sensitivity in high-fat diet–fed. AdipoRaMab treatment significantly reduced glucose levels in high-fat diet–fed, wild-type mice at each time point during the ITT period and in the AUC analysis (Fig. 4, A and B); in addition, to demonstrate this effect of AdipoRaMab treatment, analysis of the area of the curve (AOC) was performed with adjustment for the value of zero (*27*). AdipoRaMab treatment significantly increased the AOC (Fig. 4C), while this effect was obliterated in the R1·R2 DKO mice (Fig. 4, D to F), suggesting that AdipoRaMab improves insulin sensitivity via AdipoR.

### AdipoRaMab activates AdipoR pathways

**Fig. 5.**
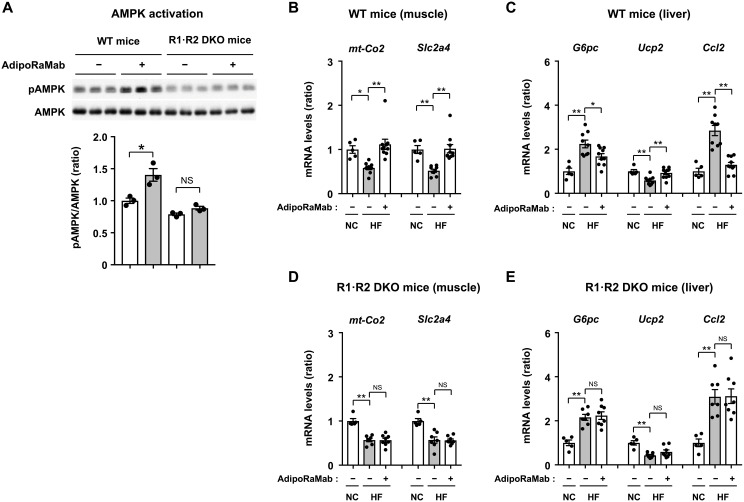
AdipoRaMab activated AdipoR1-AMPK pathway in the skeletal muscle and AdipoR1-AMPK and AdipoR2-PPARα pathway in the liver of high-fat diet–fed mice. AMPK phosphorylation at Thr^172^ (**A**) in the skeletal muscle of HF diet–fed WT and R1·R2 DKO mice, treated for 10 min after intravenous injection of AdipoRaMab (10 mg/kg body weight). *mt-Co2* and *Slc2a4* mRNA levels (**B** and **D**) in the skeletal muscle (Muscle) and *G6pc*, *Ucp2*, *Ccl2* mRNA levels in the liver (**C** and **E**) of NC- or HF diet–fed WT and R1·R2 DKO, treated four times with AdipoRaMab or isotype control Mab at a once-weekly dose of 10 mg/kg body weight from 4 weeks after initiation of an HF diet onward. AMPK phosphorylation at Thr^172^ was calculated as the ratio of the expression of phosphorylated AMPKα to that of AMPKα and quantified by Western blotting. AdipoRaMab (−), isotype control Mab treatment. Results are expressed as ratios in NC-fed mice treated with isotype control Mab. All values are presented as means ± SEM. HF diet–fed WT and R1·R2 DKO mice treated with isotype control Mab or AdipoRaMab [(A), *n* = 3]. NC-fed WT or R1·R2 DKO mice treated with isotype control Mab [(B) to (E), *n* = 5]. HF diet–fed WT mice treated with isotype control Mab or AdipoRaMab [(B) and (C), *n* = 9 or 10]. HF diet–fed R1·R2 DKO mice treated with isotype control Mab or AdipoRaMab [(D) and (E), *n* = 7 or 8]. *P < 0.05 and **P < 0.01 compared with isotype control Mab or as indicated. *P* values were determined using unpaired two-tailed *t* tests (A) or ANOVA followed by the Tukey-Kramer multiple comparison test [(B) to (E)].

Next, we examined the skeletal muscle and liver for AdipoR pathway activation with AdipoRaMab, with reference to the previously reported adiponectin/AdipoR pathway. Adiponectin has been reported to be responsible for glucose uptake and oxidative metabolism via the AdipoR1–AMPK–PPAR-γ coactivator–1α (PGC-1α) pathway in the skeletal muscle (*12*, *28*). AdipoRaMab activated AMPK in the skeletal muscle of high-fat diet–fed, wild-type mice (Fig. 5A and fig. S5), while this effect was abolished in the R1·R2 DKO mice (Fig. 5A and fig. S5), confirming that this effect was mediated by AdipoR1 and AdipoR2. In addition, AdipoRaMab increased the expression of insulin-sensitive glucose transporter 4 (*Slc2a4*) and mitochondrial cytochrome c oxidase subunit II (*mt-Co2*), i.e., the target genes of PGC-1α, which were reduced by high-fat feeding, to a similar extent to that in normal chow–fed mice (Fig. 5B), while these effects were obliterated in the R1·R2 DKO mice (Fig. 5D), confirming that these effects were mediated by AdipoR1 and AdipoR2.

The hepatic AdipoR1-AMPK pathway has been reported to suppress gluconeogenesis-related gene expression (*12*). AdipoRaMab significantly reduced the expression of the glucose-6 phosphatase (*G6pc*) gene involved in hepatic gluconeogenesis, while it was increased by high-fat diet feeding (Fig. 5C). In addition, it has been reported that the AdipoR2-PPARα pathway increases the expression of genes involved in energy expenditure and suppresses proinflammatory cytokines in the liver (*12*). AdipoRaMab increased the expression of the uncoupling protein 2 (*Ucp2*) gene responsible for energy consumption, while it was reduced by high-fat feeding to a similar level to that in normal chow–fed mice (Fig. 5C). AdipoRaMab reduced the expression of the monocyte chemoattractant protein–1 (MCP-1) (*Ccl2*) gene in the liver, while it was increased by high-fat feeding to a similar level to that in normal chow–fed mice (Fig. 5C). Again, these effects of AdipoRaMab were obliterated in the R1·R2 DKO mice (Fig. 5E), confirming that these effects were mediated by AdipoR1 and AdipoR2.

### AdipoRaMab ameliorates inflammation in the NASH mouse model

AdipoRaMab was evaluated in a NASH mouse model mimicking NASH-associated metabolic syndrome, i.e., ob/ob mice fed a trans fat–rich, high-fat, high-fructose, and high-cholesterol (TFC) diet (*29*). In this model, severe obesity, insulin resistance, hepatic fat accumulation, and hepatic dysfunction occur over a short period of time, followed several months later by the balloon-like degeneration and cellular fibrosis of the liver seen in NASH (*29*). AdipoRaMab was evaluated for its therapeutic effect on inflammation preceding fibrosis of the liver. AdipoRaMab was administered (30 mg/kg) once weekly for a total of four doses starting 4 weeks after the start of the TFC diet (table S2). At 4 weeks after the start of the TFC diet, i.e., before antibody administration, plasma alanine aminotransferase (ALT) levels were shown to be 701 ± 35 IU/liter in the control group and 698 ± 38 IU/liter in the AdipoRaMab group, and plasma insulin levels were 4.9 ± 0.3 ng/ml in the control group and 4.7 ± 0.3 ng/ml in the AdipoRaMab group. Adiponectin plasma levels were significantly reduced 4 weeks after initiation of the TFC diet (fig. S6). Food consumption during the treatment period was shown to be comparable between the AdipoRaMab and control groups (fig. S7A), with no observable difference shown in body weight between the groups (fig. S7B).

**Fig. 6.**
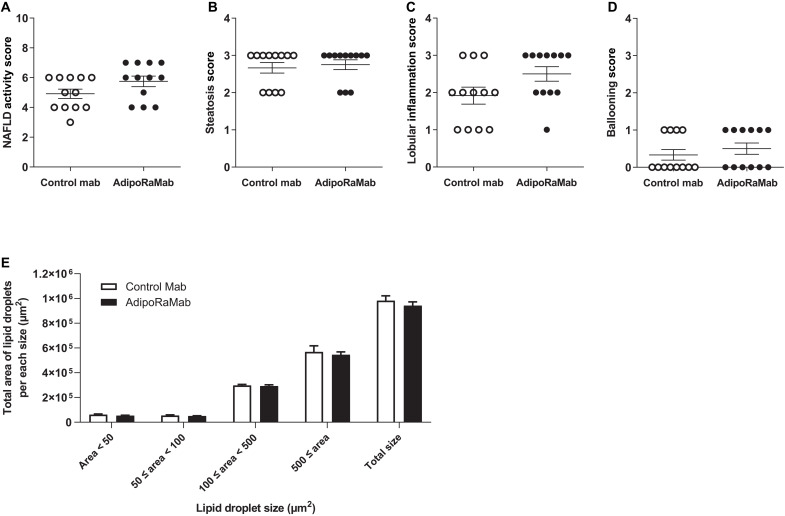
AdipoRaMab did not affect NAS and total area of lipid droplets per each size in the liver of TFC diet–fed ob/ob mice. NAS (**A**) and individual lesion scores; steatosis score (**B**), lobular inflammation score (**C**), hepatocellular ballooning score (**D**), and total area of lipid droplets per each size (**E**) in the liver of TFC diet-fed ob/ob mice, treated four times with AdipoRaMab or isotype control Mab at a once-weekly dose of 30 mg/kg body weight from 4 weeks after initiation of a TFC diet onward. Each parameter was measured using images of the hematoxylin and eosin–stained liver sections. All values were presented as means ± SEM. *n* = 12. The statistical analysis between the two groups was performed using the Wilcoxon test for (A) to (D) using unpaired two-tailed *t* test for (E), and there were no significant differences in either variable.

**Fig. 7.**
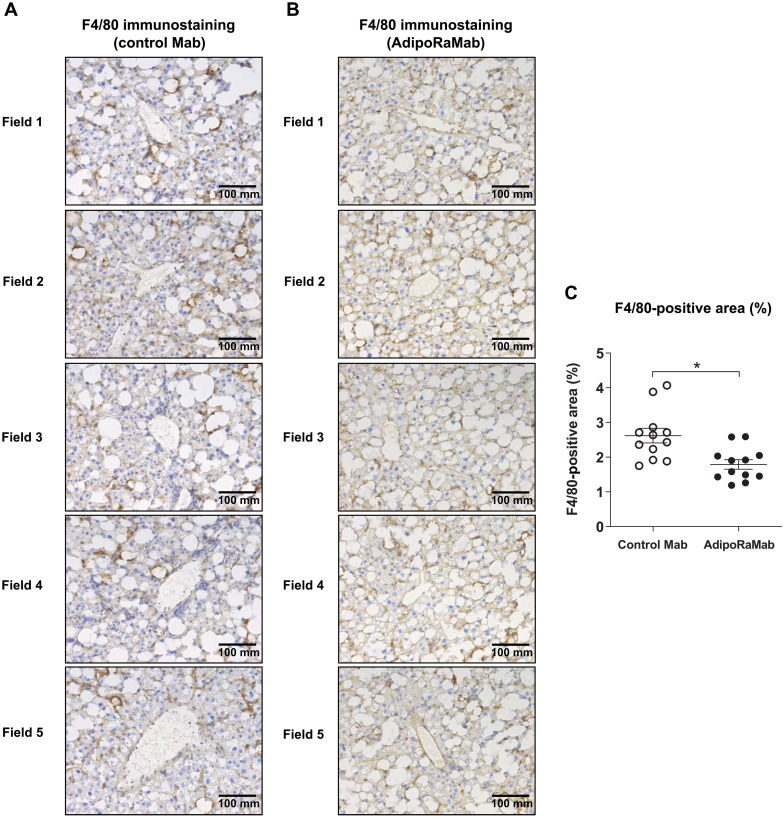
AdipoRaMab suppressed Kupffer cell/macrophage infiltration in the liver of TFC diet–fed ob/ob mice. F4/80-immunostained images of a representative single mouse from TFC diet–fed ob/ob mice, treated four times with isotype control Mab (**A**) or AdipoRaMab (**B**) at a once-weekly dose of 30 mg/kg body weight from 4 weeks after initiation of a TFC diet onward. The F4/80-positive area (%) (**C**) represents the mean of five fields derived from each mouse. All values are presented as means ± SEM. *n* = 12. *P < 0.05 compared with isotype control Mab (unpaired two-tailed *t* tests).

**Fig. 8.**
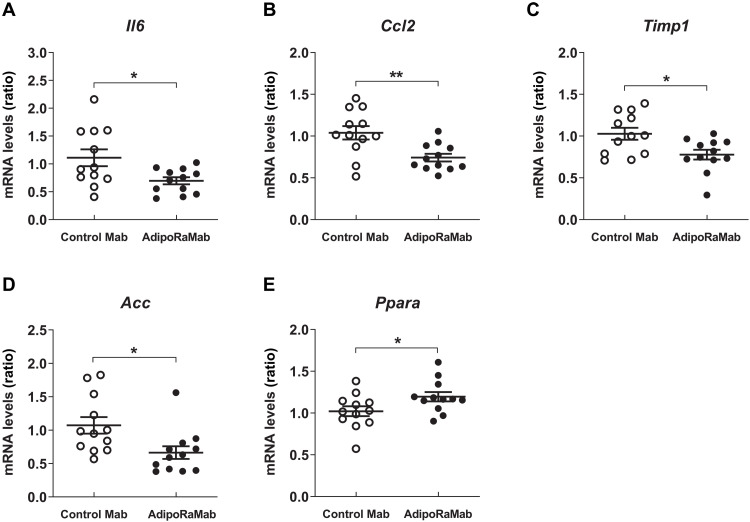
AdipoRaMab suppressed the expression of molecules involved in inflammation, fibrosis and fatty acid synthesis, and PPARα in the liver of TFC diet-fed ob/ob mice. *Il6* (**A**), *Ccl2* (**B**), *Timp1* (**C**), *Acc* (**D**), and *Ppara* (**E**) mRNA levels in the liver of TFC diet–fed ob/ob mice, treated four times with AdipoRaMab or isotype control Mab at a once-weekly dose of 30 mg/kg body weight from 4 weeks after initiation of a TFC diet onward. Results are expressed as ratios in mice treated with isotype control Mab. All values are presented as means ± SEM. *n* = 12. *P < 0.05 and **P < 0.01 compared with isotype control Mab (unpaired two-tailed *t* tests).

In the nonalcoholic fatty liver disease (NAFLD) activity score (NAS), a histologic scoring of the extent of three lesions, i.e., steatosis, lobular inflammation, and hepatocellular ballooning, there was no significant difference between the control antibody group and the AdipoRaMab group (Fig. 6, A to D). To quantitatively evaluate the effect on hepatic steatosis, the total areas of lipid droplets per each size was measured using hematoxylin and eosin (H&E)–stained images. Total area was comparable between the AdipoRaMab and control groups for all sizes (Fig. 6E). In addition, to further investigate the effect on inflammation, Kupffer cell/macrophage infiltration was evaluated by measuring the area covered by F4/80-positive cells. The area was significantly smaller in the AdipoRaMab group than in the control group (Fig. 7, A to C, and fig. S8). The gene expression analysis of the liver showed that the expression of the inflammation-related factors interleukin 6 (*Il6*) and MCP-1 (*Ccl2*) was significantly lower in the AdipoRaMab group than in the control group (Fig. 8, A and B). Furthermore, the expression of tissue inhibitor of metalloproteinases-1 (*Timp1*), an antifibrinolytic factor, was significantly lower in the AdipoRaMab group than in the control group (Fig. 8C). These results suggested that AdipoRaMab suppressed liver Kupffer cell/inflammatory macrophage infiltration and activation. In the gene analysis of the liver, the expression of acetyl-CoA carboxylase1 (*Acc*), a molecule downstream of AMPK activation, was significantly lower in the AdipoRaMab group than in the control group (Fig. 8D). In addition, the expression of PPARα (*Ppara*) in the liver was significantly higher in the AdipoRaMab group than in the control group (Fig. 8E). Serum levels of insulin, glucose, triglycerides, and total cholesterol were comparable between the AdipoRaMab and control groups (Table 1). These observations suggested that AdipoRaMab suppresses Kupffer cell/inflammatory macrophage infiltration and activation by activating signaling downstream of AdipoRs in the liver.

**Table 1.** AdipoRaMab did not affect plasma parameters in TFC diet–fed ob/ob mice. Each parameter was measured in the plasma of 8-week TFC diet–fed ob/ob mice. AdipoRaMab (*n* = 12) or an isotype control Mab (*n* = 12) was administrated subcutaneously at a once-weekly dose of 30 mg/kg body weight from 4 weeks after initiation of a TFC diet onward. All values were presented as means ± SEM. No significant differences were noted in any of the parameters evaluated between the groups (unpaired two-tailed *t* tests). TFC, trans fat–rich, high-fat, high-fructose, and high-cholesterol. AST, aspartate aminotransferase; ALT, alanine aminotransferase.

Parameters	Isotype control Mab	AdipoRaMab
ALT (IU/liter)	960 ± 94	1,099 ± 80
AST (IU/liter)	606 ± 54	697 ± 48
Glucose (mg/dl)	266 ± 12	250 ± 14
Triglyceride (mg/dl)	49 ± 4	44 ± 4
Total cholesterol (mg/dl)	380 ± 8	385 ± 9
Insulin (ng/ml)	5.6 ± 0.8	7.9 ± 1.9

## DISCUSSION

In this study, we acquired the long-acting AdipoR agonist antibody shown to improve glucose intolerance and insulin resistance, as well as NASH. This is also the AdipoR agonist antibody with potential for use in humans. Previously, Fc fusion single chain antibodies (scFv-Fcs), which bind to the C-terminal peptide of AdipoR and increase AMPK phosphorylation, were obtained from phage libraries (*30*). Given the pharmacological issue with these scFv-Fcs, i.e., a decreased blood half-life as suggested by their daily dosing, however, it was ensured that the agonist antibody had a blood half-life of 75 hours (table S2) comparable to that of endogenous mouse IgG antibodies. Generally, the blood half-life of endogenous human IgG is shown to be about four times longer than that of mouse IgG. Thus, a successfully humanized mouse agonist IgG antibody may exert a therapeutic effect even with its monthly administration, thereby not only resolving issues associated with oral therapy for type 2 diabetes, e.g., low medication adherence, but also enhancing treatment efficacy. In addition, given that antibodies raised against the linear epitopes were deemed unlikely to activate AdipoR1 and AdipoR2, we did not investigate the ability of commercially available antibodies to trigger AdipoR signaling.

AdipoRaMab exhibited effects reported for adiponectin (*6*, *7*), i.e., AMPK phosphorylation at C2C12 myotubes, and improved glucose intolerance and insulin resistance in mice with high-fat diet–induced obesity (Figs. 2 and Fig. 3). Notably, treatment with this antibody also resulted in lower blood glucose levels in these mice despite their lower plasma insulin levels than in high-fat diet–fed in the fasting state and during the OGTT, suggesting improved insulin sensitivity in these mice (Fig. 3). AdipoRaMab activated AMPK and increased the expression of genes involved in glucose uptake and oxidative metabolism reduced by high-fat feeding in the skeletal muscle (Fig. 5). In the liver, AdipoRaMab not only reduced the expression of genes involved in hepatic gluconeogenesis, which was found to have been increased by high-fat diet feeding, but also increased the expression of genes involved in energy consumption, which was found to have been reduced by high-fat feeding (Fig. 5). These effects of AdipoRaMab were obliterated in the R1·R2 DKO mice (Fig. 5). Thus, AdipoR activation by AdipoRaMab in the skeletal muscle and liver may have improved high-fat diet–induced glucose intolerance and insulin resistance through improved glucose and energy metabolism, which were found to have been reduced by high-fat diet. These mechanisms of action have been previously reported as closely mimicking those of adiponectin as mediated by AdipoR (*12*, *28*), suggesting the successful acquisition of antibodies mimicking adiponectin in this study. At the same time, this means that AdipoRaMab improves glucose intolerance and insulin resistance by binding to AdipoR in vivo.

Given that insulin resistance is shown to be strongly associated with NASH (*19*), AdipoRaMab may also be expected with its insulin-sensitizing effects to prevent NASH. In mice with high-fat diet–induced obesity, AdipoRaMab markedly suppressed proinflammatory cytokine genes in the liver, suggesting that it may protect against inflammation through its direct anti-inflammatory action. We also evaluated its therapeutic potential for NASH in a model in which severe obesity, insulin resistance, hepatic fat accumulation, and hepatic dysfunction occur over a short period of time (*29*). Despite the severe condition, AdipoRaMab also significantly suppressed not only Kupffer cell/macrophage infiltration but also the gene expression of inflammation-related factors, such as *Il6* and *Ccl2* in the liver (Figs. 7 and 8). The mean F4/80-positive area was 1.2% in the normal chow–fed mice (fig. S8) and was 2.6% in the TFC–high-fat diet–fed mice for the same period as Fig. 7. Given these results, the effect size of AdipoRaMab was not deemed small. Furthermore, AdipoRaMab suppressed the gene expression of the antifibrinolytic factor *Timp1* (Fig. 8), a factor known to be induced by IL-6 (*31*). However, no improvements were observed in plasma parameters (Table 1) and NAS (Fig. 6). Therefore, AdipoRaMab may not be deemed a promising therapeutic option for severe NASH. By using two in vivo models of mild and severe metabolic derangement in this study, AdipoRaMab was thus shown to have the potential to provide a prophylactic approach for NASH.

AdipoRaMab is now being humanized for human use. In this process, achieving a relative improvement in its activity through modification of its complementarity-determining regions is also an option. Given that decreased plasma adiponectin levels have also been implicated in other diseases, e.g., cardiovascular diseases (*32*, *33*), cancer (*34*), scleroderma (*35*), and endometriosis (*36*), the indications for AdipoRaMab are expected to extend beyond diabetes and NASH.

## MATERIALS AND METHODS

### Mice

Animals were housed in cages and maintained on a 12-hour light/12-hour dark cycle. Room temperature was controlled at 22° ± 3°C, with 50 ± 20% humidity. Experimental protocols concerning the use of laboratory animals were reviewed and approved by the Institutional Animal Care and Use Committee.

### Generation of anti-AdipoR monoclonal antibodies

Three types of AdipoR KO mice, i.e., R1 KO, R2 KO, or R1·R2 DKO mice (*12*), were subcutaneously immunized once a week with NS0 cells stably expressing AdipoR1 or AdipoR2. These cells were maintained in Dulbecco’s modified Eagle’s medium (l-glutamine-free) (Invitrogen, 11960-044) supplemented with 8.8% inactivated fetal bovine serum (FBS), 1.9% glutamine synthetase supplement (SAFC, 58672C), penicillin (88 U/ml), and streptomycin (88 μg/ml) and were peeled off with Accutase (Innovative Cell Technologies, AT104).

When serum antibody titers increased, the specific binding of the antibodies to AdipoR1 or AdipoR2 was detected by flow cytometry using CHO cells stably expressing AdipoR1 or AdipoR2. Splenocytes were fused with mouse myeloma cells (SP 2/0-Ag 14, ECACC 8507240) using 50% polyethylene glycol. Fused cells were seeded onto a 96-well plate in GIT medium (FUJIFILM Wako Pure Chemical, #396-00511) containing hypoxanthine-aminopterin-thymidine media supplement (Sigma-Aldrich, #H0262), 1% BM Condimed H1, and hybridoma cloning supplement (Roche Applied Science, #11088947001). Hybridoma clones producing anti-AdipoR antibodies were screened and cloned using the same detection method as described for serum antibody titers.

The anti-AdipoR antibody thus obtained was purified from hybridoma cultures containing FBS using an anti-mouse IgG- or IgM-immobilized column. The mouse recombinant antibody was generated using the antibody sequence identified from the hybridoma and was produced in mammalian cells. For cultures grown in serum-free medium, it was purified using protein A.

### C2C12 cells

C2C12 cells were used after myogenic differentiation. Induction of myogenic differentiation was carried out according to a method described previously (*7*). By day 7, the cells were shown to have differentiated into multinucleated contracting myotubes.

### Western blot analysis

Phosphorylation and protein levels of αAMPK (*37*–*40*) were determined. Western blot analyses were performed with anti-phosphorylated AMPK (Cell Signaling Technology, #2535) and anti-αAMPK (1:1000; Cell Signaling Technology, #2532) antibodies. To study AMPK phosphorylation in vivo, AdipoRaMab was intravenously injected into mice at dose of 10 mg/kg body weight through an inferior venacava catheter (*7*, *15*). Uncropped Western blot images are shown in figs. S6 and S7. Recombinant human adiponectin was purchased from Enzo Life Sciences (ALX-522-063-C050).

### Oral glucose and ITTs

The tests used male C57BL/6 mice (CLEA Japan Inc.) or R1·R2 DKO mice fed a high-fat diet 32 consisting of 32% fat (CLEA Japan Inc.) for 8 weeks and normal chow–fed mice (CE-2, CLEA Japan Inc.). After 4-week dietary intervention, mice were treated four times with antibodies (AdipoRaMab or the isotype control Mab; Medical & Biological Laboratories Co. Ltd) at a once-weekly dose of 10 mg/kg body weight. The tests were carried out according to a previously published method (*6*, *7*). The areas under the glucose and insulin curves were calculated by multiplying the cumulative mean height of the glucose values and insulin values, respectively, by time (*6*, *12*). The AOC was calculated by subtracting the value at 0 min from the value at each time point (*27*). Insulin resistance index was calculated from the product of the areas under the glucose and insulin curves × 10^−2^ in the glucose tolerance test. The results are expressed as the percentage of the value for control mice (*6*, *12*).

### Quantitative gene expression analysis using real-time polymerase chain reaction

Total RNA was prepared from cells or tissues with TRIzol (Invitrogen) or the Maxwell simplyRNA Purification Kit (Promega) according to the manufacturer’s instructions. A real-time polymerase chain reaction (PCR) method was used to quantify the mRNAs (*11*, *12*, *28*), with slight modifications. The real-time PCR was performed using specific TaqMan Gene Expression Assays (table S3; Applied Biosystems) and cyclophilin (forward primer: 5′-GGTCCTGGCATCTTGTCCAT-3′; reverse primer: 5′-CAGTCTTGGCAGTGCAGATAAAA-3′; probe: 5′-CTGGACCAAACACAAACGGTTCCCA-3′ as internal control in Fig. 5 (B to E).

### Preparation of the NASH mice model

Male C57BL/6J Ham Slc-ob/ob mice (age 7 weeks, ob/ob mice) were obtained from SLC Japan Inc. (Tokyo, Japan). The model was generated as described previously (*29*). Briefly, ob/ob mice were fed on a high-fat (40 kcal %, containing 30 kcal% trans fat), high-fructose (20 kcal %), and high-cholesterol [2% (w/w)] (TFC) diet (D09100301, Research Diet, USA) ad libitum from the age of 8 weeks until the end of the evaluation.

### AdipoRaMab administration to NASH mice model

After 4-week dietary intervention, mice were treated with antibodies (AdipoRaMab or the isotype control Mab; Medical & Biological Laboratories Co. Ltd) at a once-weekly dose of 30 mg/kg body weight. The mice were grouped according to body weight, plasma insulin levels, and plasma ALT levels. Body weight and calorie intake were measured during the treatment period. After 4 weeks, mice were euthanized, and their liver and plasma samples were collected for histological, gene expression, and plasma biochemical analyses.

### Liver histological analysis

Liver samples were collected from the left lateral lobe; fixed in 10% formalin, paraffin-embedded; and sliced into 4-μm sections. Tissue sections were stained with H&E, observed using a bright-field microscope (Leica Microsystems). NAS was evaluated according to previous reports (*41*). The lipid droplet size was measured using the images of H&E-stained liver sections by the value module (Indica labs) of HALO with reference to a published report (*42*). Additional liver samples were embedded in the optimal cutting temperature compound, immersed in liquid nitrogen to obtain a frozen block, and sliced into 5-μm sections. To assess the development of hepatic crown-like structures, an anti-F4/80 antibody (T-2028, BMA Biomedicals, Augst, Switzerland) diluted with Block Ace (DS Pharma Biomedical, #UK-B80) was used as the primary antibody and a goat anti-rat IgG antibody (62-9520, Thermo Fisher Scientific Inc., MA, USA) diluted with Block Ace as the secondary antibody for immunohistochemical analyses. The specimens were examined using a bright-field microscope (Leica Microsystems). Photographs were taken in five fields per section in a 200× field positioned on the central vein using a charge-coupled device camera (Leica Microsystems). The photographed areas and F4/80-positive areas in each visual field were measured using ImageJ software (National Institutes of Health). Histological assessment was performed by a pathologist blinded to the study.

### Biochemical analysis of plasma

ALT, aspartate aminotransferase, glucose, triglycerides, and total cholesterol plasma levels were measured using an autoanalyzer, Hitachi 7070 (Hitachi, Tokyo, Japan). Each assay reagent was purchased from Serotec Co. Ltd. Insulin plasma levels were measured using an enzyme-linked immunosorbent assay kit for mouse insulin (Morinaga, Tokyo, Japan). All measurements were performed according to the manufacturer’s instructions for each kit used.

### Statistical analysis

All data are expressed as means ± SEM. Differences between two groups were assessed for significance using unpaired two-tailed *t* tests. Differences in scoring data between two groups were assessed for significance using the Wilcoxon test. Data involving more than two groups were assessed for significance by analysis of variance (ANOVA) followed by the Tukey-Kramer multiple comparison test. Data including dose response in more than two groups were assessed for significance by ANOVA followed by the Dunnett’s multiple comparison test. Differences in the distribution of the two histograms were assessed for significance using the Kolmogorov-Smirnov test. The level of significance was set at 5%.
