# Supplementary material for: A monoclonal antibody activating AdipoR for type 2 diabetes and nonalcoholic steatohepatitis
Source: Sci Adv. 2023 Nov 10;9(45):eadg4216. doi: 10.1126/sciadv.adg4216 (PMC10637737; doi:10.1126/sciadv.adg4216)
Supplement: Supplementary file 1 — Figs. S1 to S8 Tables S1 to S3 [file sciadv.adg4216_sm.pdf]

Science Advances
AAAS

Supplementary Materials for

A monoclonal antibody activating AdipoR for type 2 diabetes and nonalcoholic steatohepatitis

Naomi Asahara *et al.*

Corresponding author: Toshimasa Yamauchi, tyamau@m.u-tokyo.ac.jp;
Takashi Kadowaki, t-kadowaki@toranomon.kkr.or.jp

Sci. Adv. **9**, eadg4216 (2023)
DOI: 10.1126/sciadv.adg4216

This PDF file includes:

Figs. S1 to S8
Tables S1 to S3

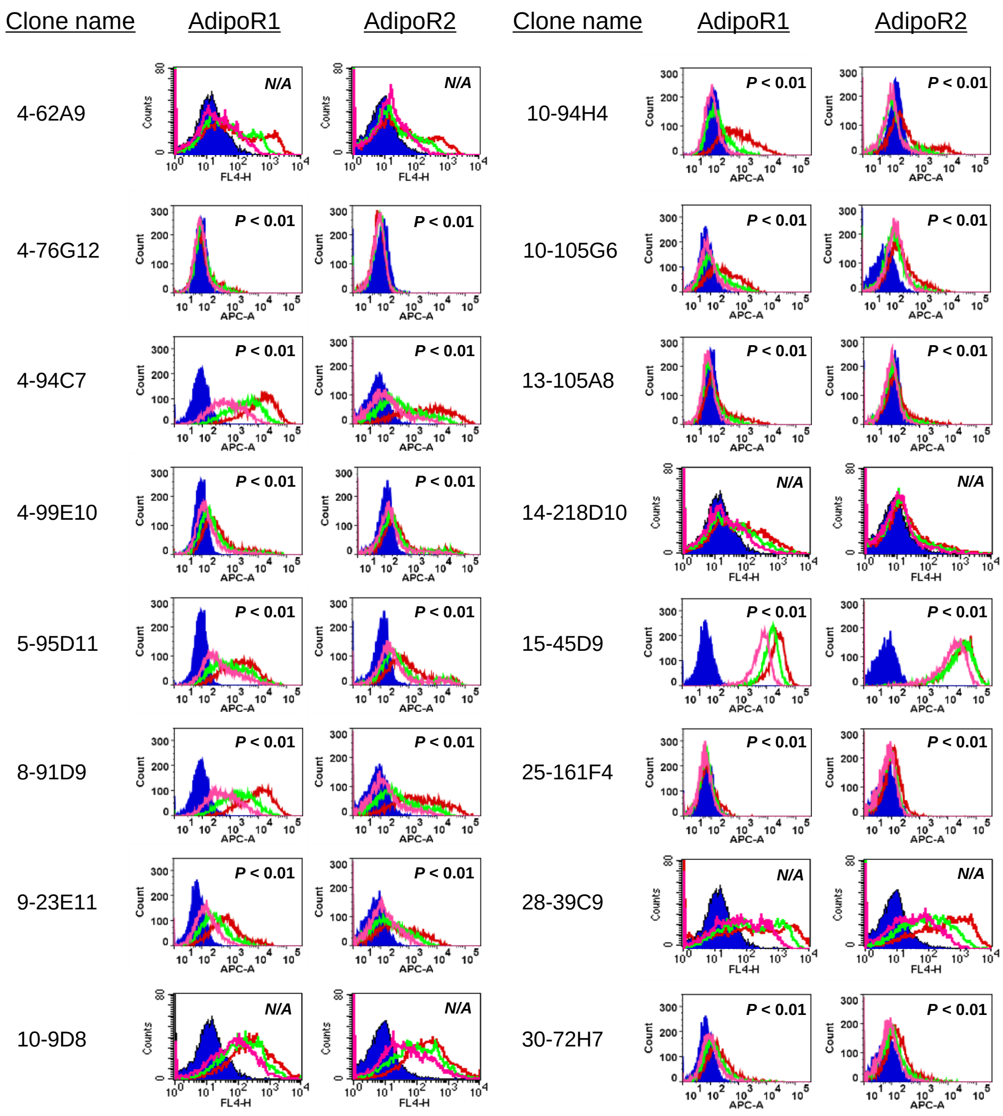

Fig. S1. Binding characteristics of anti-AdipoR antibodies shown on Table S1. Anti-AdipoR antibodies were evaluated for binding to AdipoR1 or AdipoR2 in AdipoR1- or AdipoR2-expressing cells by using flow cytometry, and the results were presented as histograms. Solid line histograms represent those for isotype control Mab (10 μg/ml). Red, anti-AdipoR antibodies 10 μg/ml; green, Anti-AdipoR antibodies 3 μg/ml; and pink, Anti-AdipoR antibodies 1 μg/ml. Statistical analyses were performed using the Kolmogorov-Smirnov test with the level of significance set at 5%. N/A, not applicable. Histogram for the antibody of clone name: 4-76G12 shown to be significantly toward a lower fluorescence intensity than that for the control antibody.

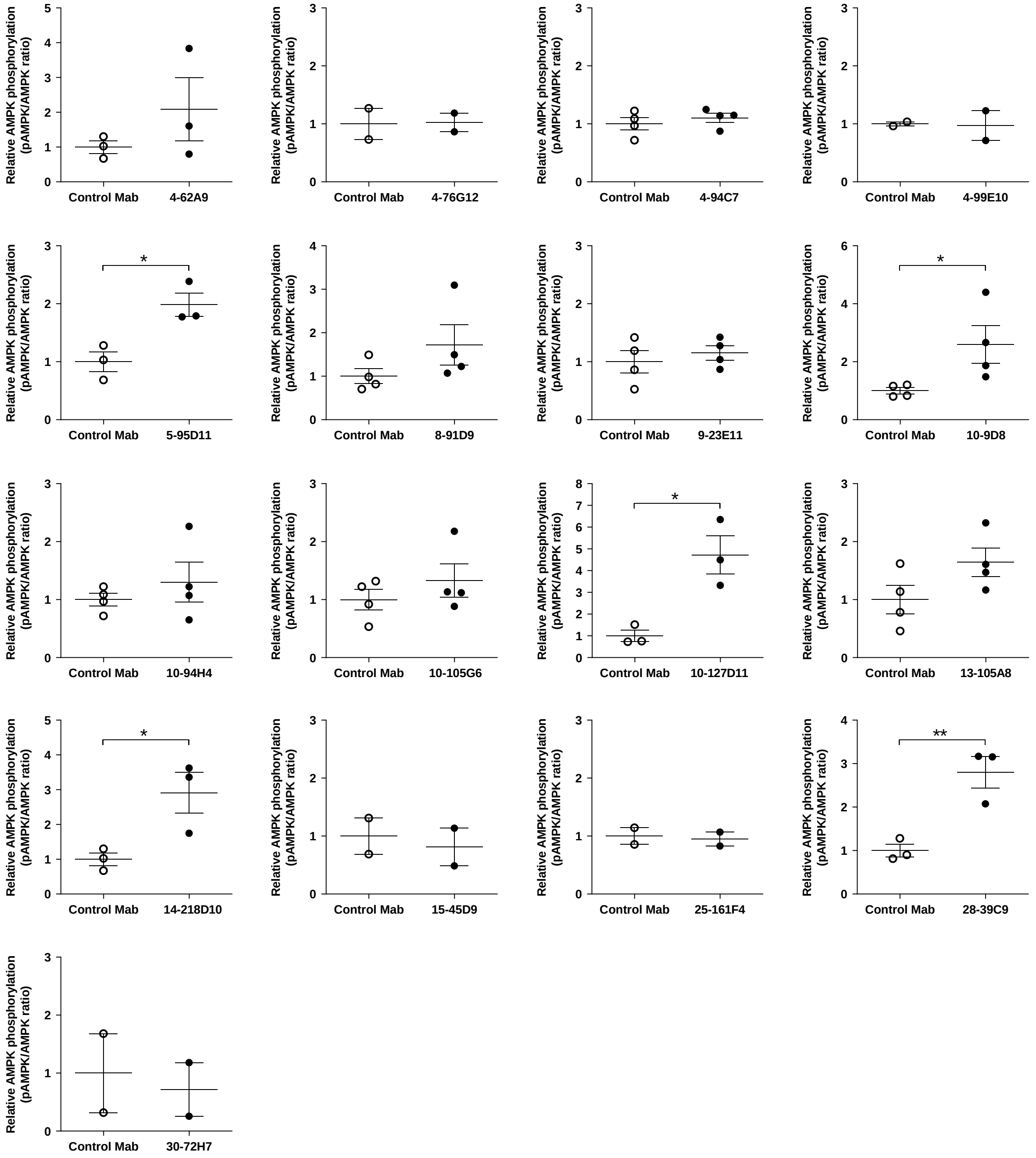

Fig. S2. AMPK activation of anti-AdipoR antibodies shown on Table S1. C2C12 myotubes were evaluated for AMPK activation with each antibody in multiple independent experiments. C2C12 myotubes were treated with an isotype control Mab (10 µg/ml) or anti-AdipoR antibody (10 µg/ml) for 5 min. For each experiment, phosphorylated AMPKα and AMPKα were quantified by western blot, and the ratio of phosphorylated AMPKα/AMPKα was calculated. The results are expressed as relative ratios of means for anti-AdipoR/control antibodies. All values are presented as mean ± SEM, n = 2-4. *P < 0.05, **P < 0.01 vs. isotype control Mab (unpaired two-tailed t-tests).

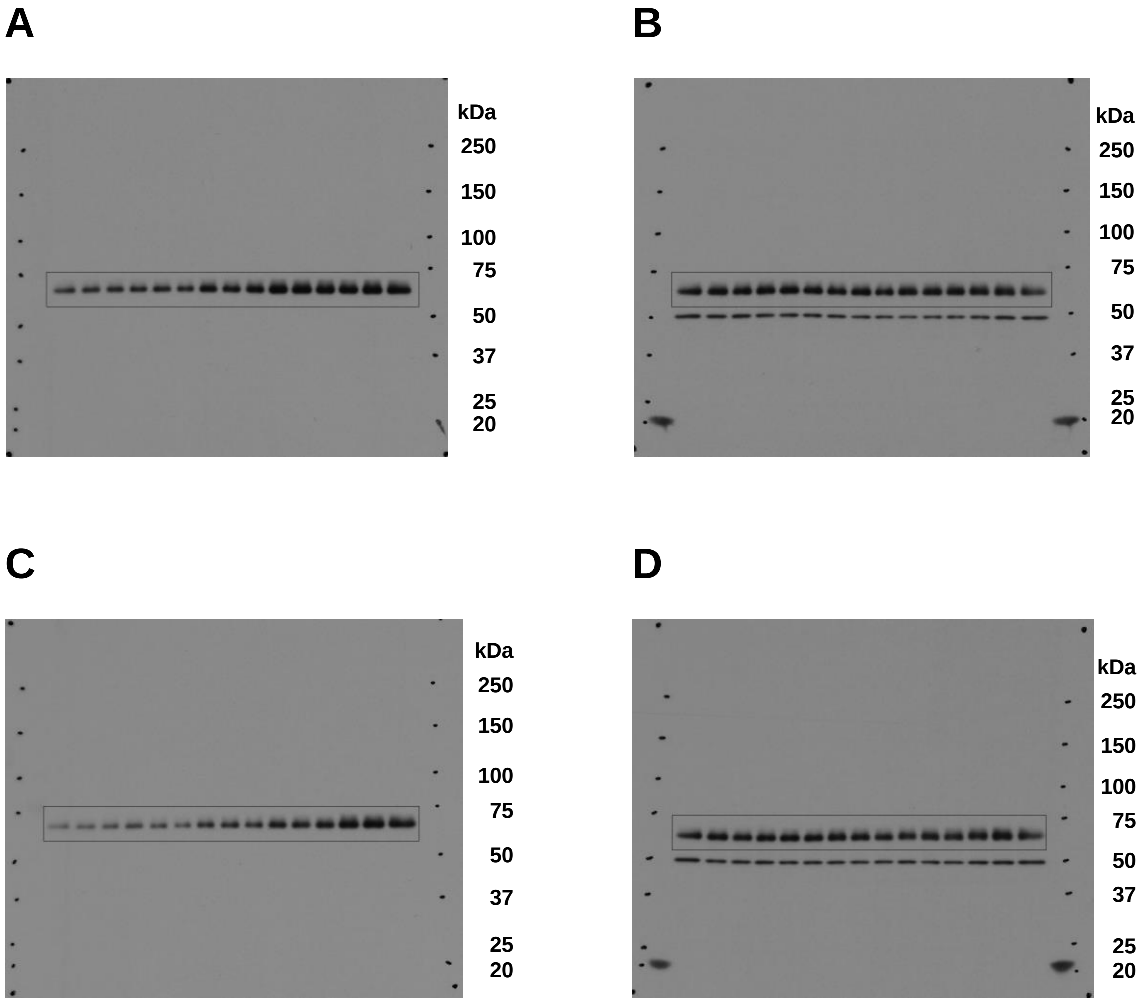

Fig. S3. Full-length, unprocessed western blots of each experiment shown in Fig. 2 Blots at Thr172 of AMPK phosphorylation (A) and AMPK (B) in Fig. 2A. Blots at Thr172 of AMPK phosphorylation (C) and AMPK (D) in Fig. 2B .

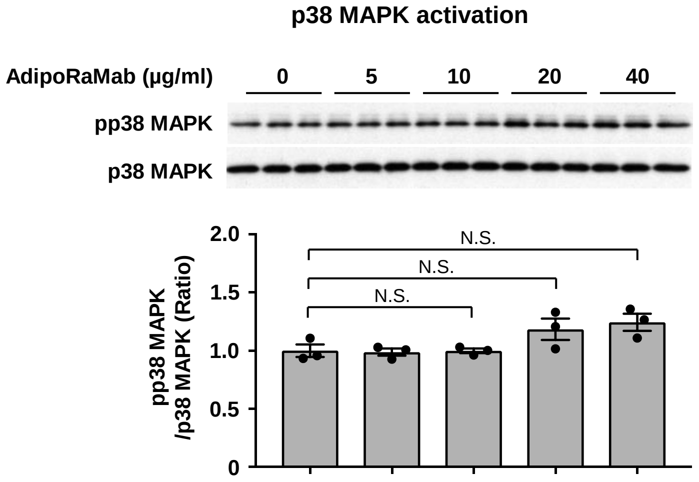

Fig. S4. AdipoRaMab did not affect on p38MAPK phosphorylation in C2C12 myotubes. Phosphorylation and amount of p38MAPK in C2C12 myotubes after their myogenic differentiation. C2C12 myotubes were treated with or without AdipoRaMab at the concentration indicated for 10 min. p38MAPK phosphorylation was calculated as the ratio of the expression of phosphorylated p38MAPK (pp38MAPK) to that of p38MAPK and quantified by western blotting. Results are expressed as ratios without AdipoRaMab. All values are presented as mean ± SEM. n = 3, *P < 0.05, **P < 0.01 compared with no AdipoRaMab (ANOVA followed by the Dunnett multiple comparison test).

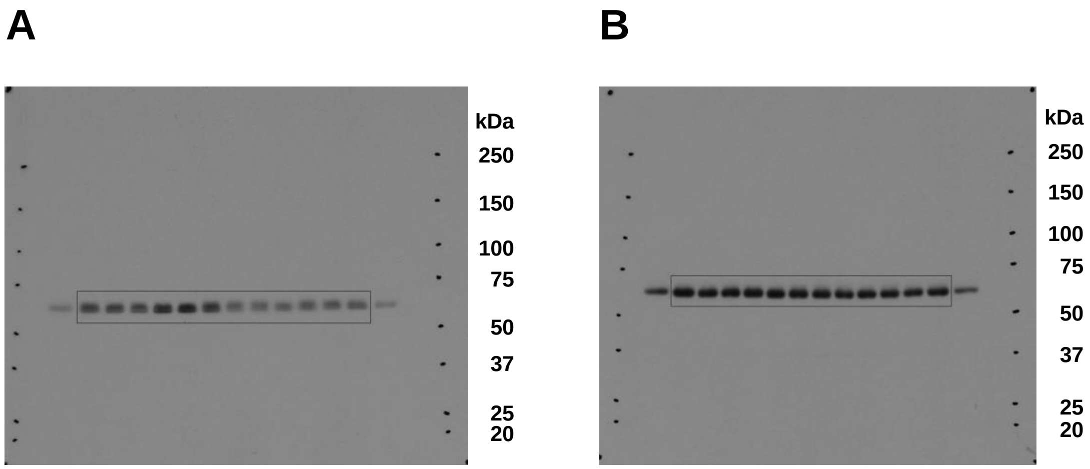

Fig. S5. Full-length, unprocessed western blots of each experiment shown in Fig. 5A Blots at Thr172 of AMPK phosphorylation (A) and AMPK (B) in Fig. 5A

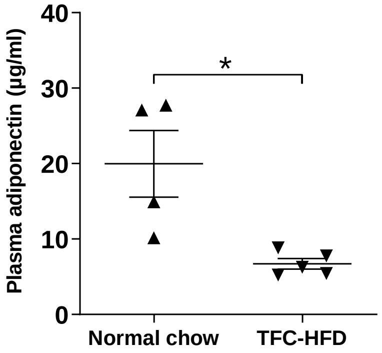

Fig. S6. Plasma adiponectin content in TFC diet-fed ob/ob mice. Ob/ob mice fed normal chow (n = 4) or TFC diet (n = 5) for 4 weeks. All values are presented as mean ± SEM. *$P < 0.05$ compared with normal chow (unpaired two-tailed t-tests).

A B

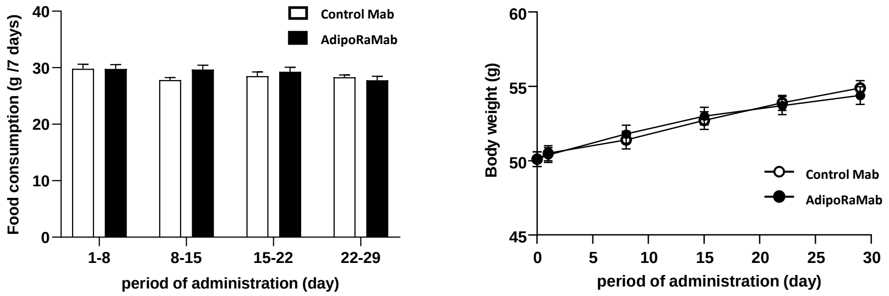

Fig. S7. AdipoRaMab did not affect body weight and food consumption during treatment period in TFC diet-fed ob/ob mice. Food consumption (A) was expressed as the sum of food consumed per week during the antibody treatment period. Body weight (B) was measured before and every other week after initiation of AdipoRaMab (n = 12) or isotype control Mab (n = 12). All values were presented as mean ± SEM. The statistical analysis between the two groups was performed using the t-test, and there were no significant differences in either variable.

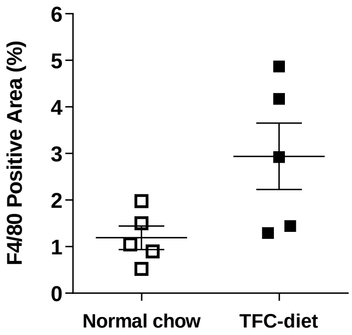

Fig. S8. F4/80-positive area (%) in liver of normal chow-fed or TFC diet-fed ob/ob mice. The F 4/80-positive area (%) represents the mean of five fields derived from each mouse. The ob/ob mice were fed TFC diet for 4 weeks. All values are presented as mean ± SEM. n = 5

Table S1. Characterization of anti-AdipoR antibodies acquired via the immunization of AdipoR KO mice using cells stably expressing AdipoR. R1 and R2 represent NS0 cells stably expressing AdipoR1 or AdipoR2. The binding activities were evaluated by flow cytometry using CHO cells stably expressing AdipoR1 or AdipoR2 and were determined based on histograms at 10 µg/ml. +, histogram for the anti-AdipoR antibody shown to be significantly shifted toward a higher fluorescence intensity than that for the control antibody. -, histogram for the anti-AdipoR antibody shown to be otherwise. Statistical analyses were performed using the Kolmogorov-Smirnov test with the level of significance set at 5%. N/A, not applicable. AMPK activation was evaluated in C2C12 myotubes at 10 µg/ml. +, relative ratio shown to be significantly higher; -, relative ratio shown to be otherwise. Statistical analyses were performed using unpaired two-tailed t-tests with the level of significance set at 5%. For binding characteristics and AMPK activation, detailed data are provided in Fig. S1, and Fig. S2.

**Immunization**		**Hybridoma**		**Binding characteristics**		**AMPK activation**
Mice	Antigen	Clone name	Antibody Class	AdipoR1	AdipoR2	
R1 KO	R1	4-62A9	IgM, K	N/A	N/A	−
		4-76G12	IgM, K	+	−	−
		4-94C7	IgG2b, K	+	+	−
		4-99E10	IgM, K	+	+	−
		5-95D11	IgM, K	+	+	+
		8-91D9	IgG2b, K	+	+	−
		9-23E11	IgM, K	+	+	−
		10-9D8	IgM, K	N/A	N/A	+
		10-94H4	IgG2a, K	+	+	−
		10-105G6	IgM, K	+	+	−
		10-127D11	IgG2b, K	+	+	+
R2 KO	R2	13-105A8	IgM, K	+	+	−
		14-218D10	IgM, K	N/A	N/A	+
		15-45D9	IgM, K	+	+	−
R1·R2 DKO	R1	25-161F4	IgM, K	+	+	−
	R2	28-39C9	IgM, K	N/A	N/A	+
		30-72H7	IgM, K	+	+	−

Table S2. PK parameters in TFC diet-fed ob/ob mice following subcutaneous administration of AdipoRaMab at a dose of 10 mg per kg body weight (Non-compartmental analysis). AUC, area under the blood concentration-time curve; t, time after administration (hour)

$AUC_{(0\text{-}t)}$ µg/ml x hour	$AUC_{(0\text{-}infinity)}$ µg/ml x hour	$t_{1/2}$ (hour)
2,772	6,433	75

Table S3. TaqMan Gene Expression Assays

Gene Symbol	Gene Name	TaqMan Assay ID
Acaca (*ACC*)	acetyl-CoA carboxylase 1	Mm01304257_m1
Actb	beta-actin	Mm00607939_s1
Ccl2	monocyte chemotactic protein 1	Mm00441242_m1
G6pc	glucose-6 phosphatase	Mm00839363_m1
Il6	interleukin 6	Mm00446190_m1
mt-Co2	mitochondrial cytochrome c oxidase subunit II	Mm03294838_g1
Ppara	peroxisome proliferator-activated receptor alpha	Mm00440939_m1
Slc2a4	insulin-sensitive glucose transporter 4	Mm00436615_m1
Timp1	tissue metallopeptidase inhibitor 1	Mm00441818_m1
Ucp2	uncoupling protein 2	Mm00495907_g1
